# Recapitulating the Lateral Organization of Membrane Receptors at the Nanoscale

**DOI:** 10.1021/acsnano.3c00683

**Published:** 2023-05-18

**Authors:** Seyed R. Tabaei, Marcos Fernandez-Villamarin, Setareh Vafaei, Lorcan Rooney, Paula M. Mendes

**Affiliations:** † School of Chemistry and Chemical Engineering, Queen’s University Belfast, Stranmillis Road, Belfast, BT9 5AG, U.K.; § School of Chemical Engineering, University of Birmingham, Edgbaston, Birmingham, B15 2TT, U.K.

**Keywords:** nanoclusters, receptor crowding, membrane receptor, surface molecular imprinting, multivalent interaction

## Abstract

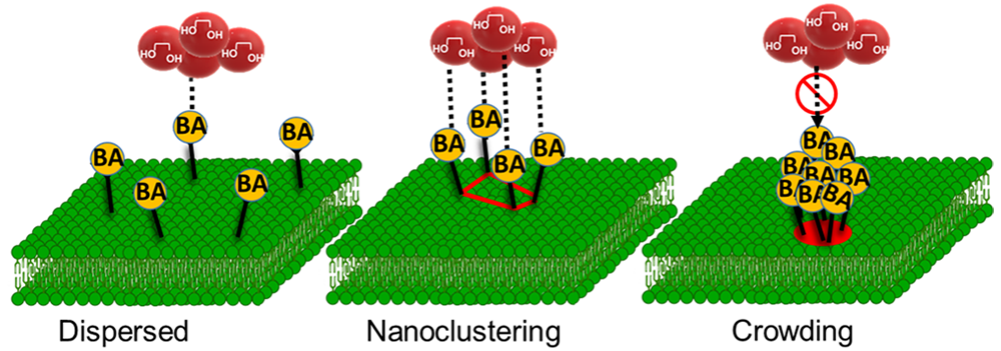

Many cell membrane functions emerge from the lateral presentation of membrane receptors. The link between the nanoscale organization of the receptors and ligand binding remains, however, mostly unclear. In this work, we applied surface molecular imprinting and utilized the phase behavior of lipid bilayers to create platforms that recapitulate the lateral organization of membrane receptors at the nanoscale. We used liposomes decorated with amphiphilic boronic acids that commonly serve as synthetic saccharide receptors and generated three lateral modes of receptor presentation—random distribution, nanoclustering, and receptor crowding—and studied their interaction with saccharides. In comparison to liposomes with randomly dispersed receptors, surface-imprinted liposomes resulted in more than a 5-fold increase in avidity. Quantifying the binding affinity and cooperativity proved that the boost was mediated by the formation of the nanoclusters rather than a local increase in the receptor concentration. In contrast, receptor crowding, despite the presence of increased local receptor concentrations, prevented multivalent oligosaccharide binding due to steric effects. The findings demonstrate the significance of nanometric aspects of receptor presentation and generation of multivalent ligands including artificial lectins for the sensitive and specific detection of glycans.

## Introduction

Cell membrane receptors respond to ligands with high sensitivity and plasticity. However, these crucial high sensitivities cannot be attributed solely to the affinity of individual receptors for their ligands. For example, T cells have low-affinity receptors (T-cell antigen receptors) that however are very sensitive to their antigen peptide ligands.^1,2^ The high level of sensitivity can be achieved by grouping membrane receptors together into clusters, thereby raising receptor density at the membrane interface, as the cooperative interaction of clustered receptors with ligands can lead to high functional affinity (avidity). Accordingly, the high affinity can be explained by the random concentration of receptors in the form of clusters and thus the stoichiometry and cooperativity of the ligand–receptor interactions. Such clustering can be achieved, for instance, by partitioning membrane receptors in the cholesterol- and sphingomyelin-rich lipid raft domains. However, increasing evidence suggests that membrane receptors can be arranged into distinctly sized nanoclusters in non-raft domains with a high degree of lateral organization.^3,4^

The impact of receptor number density and lateral organization on the formation of more efficient receptor–ligand complexes is, however, not well understood. For example, while a high density of ganglioside GM1 is needed to ensure maximal binding of cholera toxin (CT) to the cell surface in order to trigger signal transduction,^5−7^ studies of the CT binding to GM1 using synthetic lipid bilayer systems showed that increasing the GM1 density weakened the CT binding, presumably due to steric effects caused by GM1 clustering. These examples show that to advance our understanding of the molecular basis of the interfacial interactions at the membrane interfaces, it is essential to study the impact of receptor nanoclustering (i.e., nanosized clusters of ordered receptors), optimal receptor presentation, and receptor crowding (domains of too densely and randomly packed receptors) on target accessibility and binding interactions.

Lipid bilayers have been extensively used as biomimetic models to study molecular interactions at lipid membrane interfaces.^8−17^ However, the mere inclusion of amphiphilic receptors in the bilayer formulations often results in a homogeneous or random distribution of receptors that fails to sufficiently resemble functional cell-membrane nanodomains. Importantly, convincing evidence suggests that binding events at the membrane interface can lead to reorganization of lipid-membrane components.^16,18^ Notably, by using polymerizable lipids, the organization of membrane components can be “arrested” via a process reminiscent of the surface molecular imprinting.^19^ In this work, by applying surface molecular imprinting and utilizing the phase behavior of lipid bilayers, we created platforms that recapitulate the lateral organization of membrane receptors at the nanoscale.

Amphiphilic boronic acids (BAs) and saccharides were used as membrane receptor and in-solution ligand models, respectively. BAs are the most commonly used recognition moieties for the synthesis of binders for *cis*-diol-containing biomolecules, such as saccharides, which contain many hydroxyl groups.^20−22^ BAs can form reversible five- or six-membered cyclic esters with 1,2- or 1,3-*cis*-diol-containing compounds, often under alkaline pH conditions.^23^ In general, the affinity of single BAs for *cis*-diols is relatively low, ranging from 10^–1^ to 10^–3^ M.^24,25^ As such, a wide range of BA-functionalized nanomaterials have been developed for the recognition, extraction, and separation of saccharides.^26−30^ Moreover, several liposome platforms containing amphiphilic boronic acid derivatives have been reported. For instance, Best et al. developed a range of boronic acid and bis-boronic acid lipids to create saccharide-responsive liposomes for drug delivery and controlled release applications.^31−33^

The multivalent and reversible interaction between the BA and oligosaccharides makes them an ideal minimalist chemical system for modeling the interaction between membrane-embedded receptors and in-solution multivalent ligands. In this regard, using the BA/saccharide model, we demonstrated the significance of the nanometric aspect of receptor presentation in general and could differentiate between prearranged nanoclusters and crowding of dispersed receptors as two modes of membrane-embedded receptor presentation. As for saccharide recognition, we developed a method to achieve a finely controlled arrangement of BA units for enhanced binding. Saccharides carry key information in biological systems, which makes them an important source of biomarkers for a wide range of diseases.^34^ However, due to their inherent diversity and complexity, selective saccharide recognition remains a difficult task. We demonstrated that the increased surface density of the BAs on the surface is not enough for the enhanced saccharide binding; rather, a controlled arrangement of the BAs is important.

## Results and Discussion

**Figure 1:**
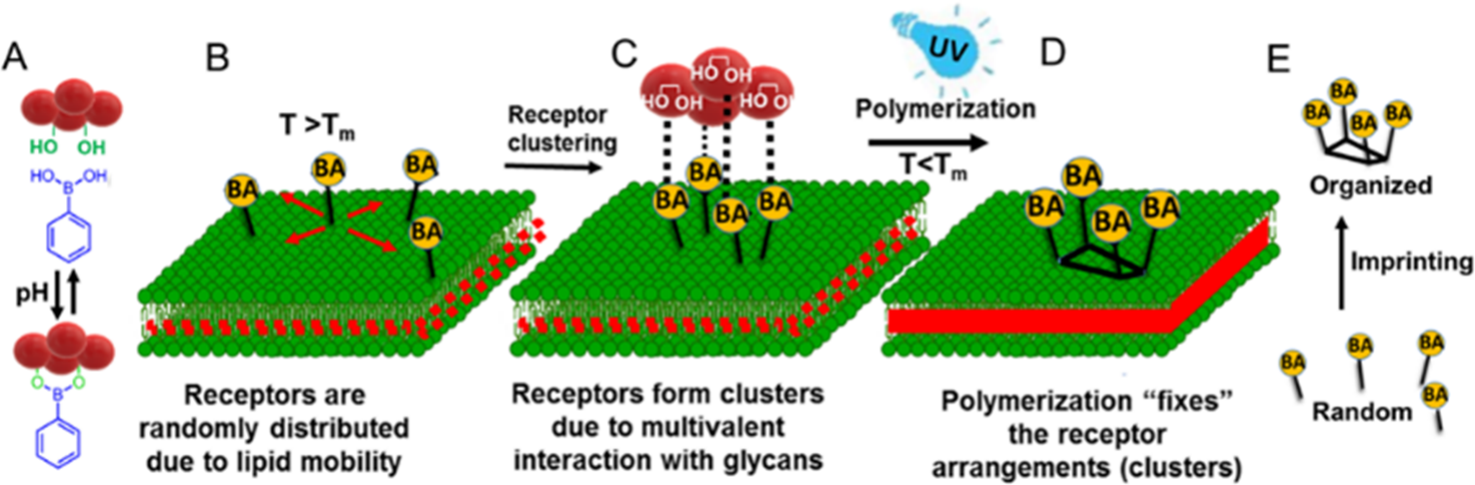
Schematic representation of the template-guided assembly of BA clusters. (A) BA covalently and reversibly binds with 1,2- or 1,3-*cis*-diols to form five- or six-membered cyclic boronic esters in alkaline solution. The cyclic esters dissociate at an acidic pH. (B) Free lateral diffusion of BA receptors in the fluid lipid membrane (*T* > *T*_m_), followed by (C) exposure to the target saccharide to form BA clusters upon multivalent interaction. Lipid mobility facilitates receptor (BA) recruitment by the multivalent ligand (oligosaccharide). (D) Polymerization of the matrix lipids at a temperature below the phase transition temperature (*T* < *T*_m_) to “freeze” the optimum arrangement of otherwise randomly distributed BA receptors. The nanoclusters on the surface of the imprinted liposomes are stabilized by photopolymerization. The polymerization step covalently connects all monomers, thereby fixing the arrangement of receptors and preventing lipid demixing. (E) Ligand-directed rearrangement of BA.

We developed a method to create nanoclusters of amphiphilic BAs at the membrane interface. The schematic presentation of the template-mediated clustering of BAs at the membrane interface is presented in Figure 1. This lipid membrane surface patterning is analogous to surface molecular imprinting in that functional monomers are cross-linked on the surfaces of a support in the presence of a template.^35−37^ The polymerizable functional amphiphiles bearing BA derivatives are incorporated into the membrane, where they are randomly distributed in the absence of a target due to free lateral diffusion of lipids. Upon exposure of the membrane to the target saccharide at a temperature above the phase transition temperature (*T*_m_) of the lipid bilayer, the freely mobile BAs reorganize to form clusters. The BAs in these clusters adopt a geometric arrangement that matches reciprocal functionalities (i.e., *cis*-diols) on the multivalent saccharide template. Finally, photopolymerization “fixes” the receptor’s (i.e., BAs) arrangement so that it is preserved after template unbinding.

**Scheme 1:**
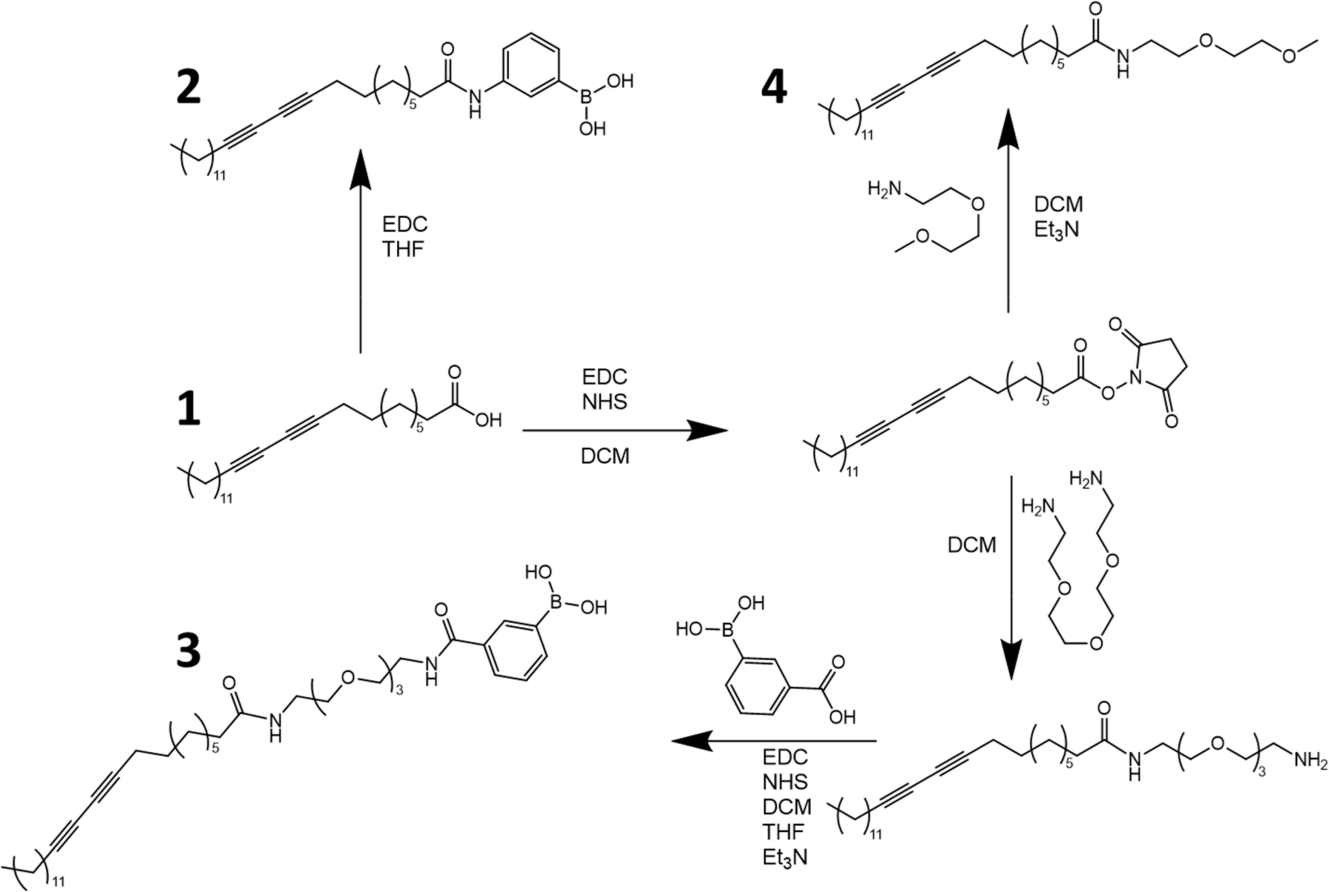
Synthesis of the PCDA derivatives. (1) PCDA, (2) PCDA-BA with no PEG linker, (3) PCDA-PEG-BA, and (4) PCDA-PEG.

Well-aligned diacetylene monomers in solution can form a variety of morphologies, including liposomes.^38^ Importantly, self-assembled amphiphilic diacetylene monomers, such as 10,12-pentacosadiynoic acid (PCDA),^19^ have been shown to polymerize in a 1,4-addition type to generate ene-yne polydiacetylene (PDA) polymers via UV irradiation (254 nm) with no catalysts or initiators.^20^ This feature of PDAs makes them very practical for imprinting purposes.^19^

We synthesized a variety of functional diacetylene monomers, in order to create photopolymerizable liposomes as a platform for the molecular imprinting of saccharides on lipid membrane surfaces. The receptor incorporates a spacer chain of four hydrophilic oligo(ethylene glycol) units to make BA more accessible to saccharides at the membrane interface. PCDA-PEG-BA monomer was obtained by direct reaction between an *N*-hydroxysuccimide (NHS) PCDA derivative with a diamino PEG and subsequent reaction of the product with 3-carboxyphenylboronic acid using *N*-ethyl-*N*′-(3-(dimethylamino)propyl)carbodiimide (EDC) and NHS. Alternatively, a receptor without PEG was also synthesized in order to test the linker effect. PCDA-BA was obtained by direct conjugation on a 3-aminophenylboronic acid to a PCDA molecule with EDC. Finally, a monomer without BA was used as the polymerizable matrix lipids in combination with the BA-functionalized monomers to construct the liposomes. PCDA-PEG was also obtained by direct reaction between the NHS PCDA derivative and (2-(2-methoxyethoxy)ethyl)amine.

Liposomes of the desired composition were prepared by sonication at 80 °C, above the phase transition temperature (*T*_m_) of PCDAs. For imprinting, the liposomes were incubated with template saccharide at this temperature at pH 10 for 1 h before storage at 4 °C for 24 h. The transparent solution was then irradiated with 254 nm UV light. The nonimprinted liposomes were prepared the same way without incubation with the template. The total lipid concentrations were typically between 0.5 and 2 mM, as higher concentrations can cause the PCDA to precipitate out of solution. A typical size distribution of liposomes is shown in Supporting Figure S1. The average diameter of the functionalized PDA liposomes was determined as 200 ± 59.9 nm depending on the lipid composition.

**Figure 2:**
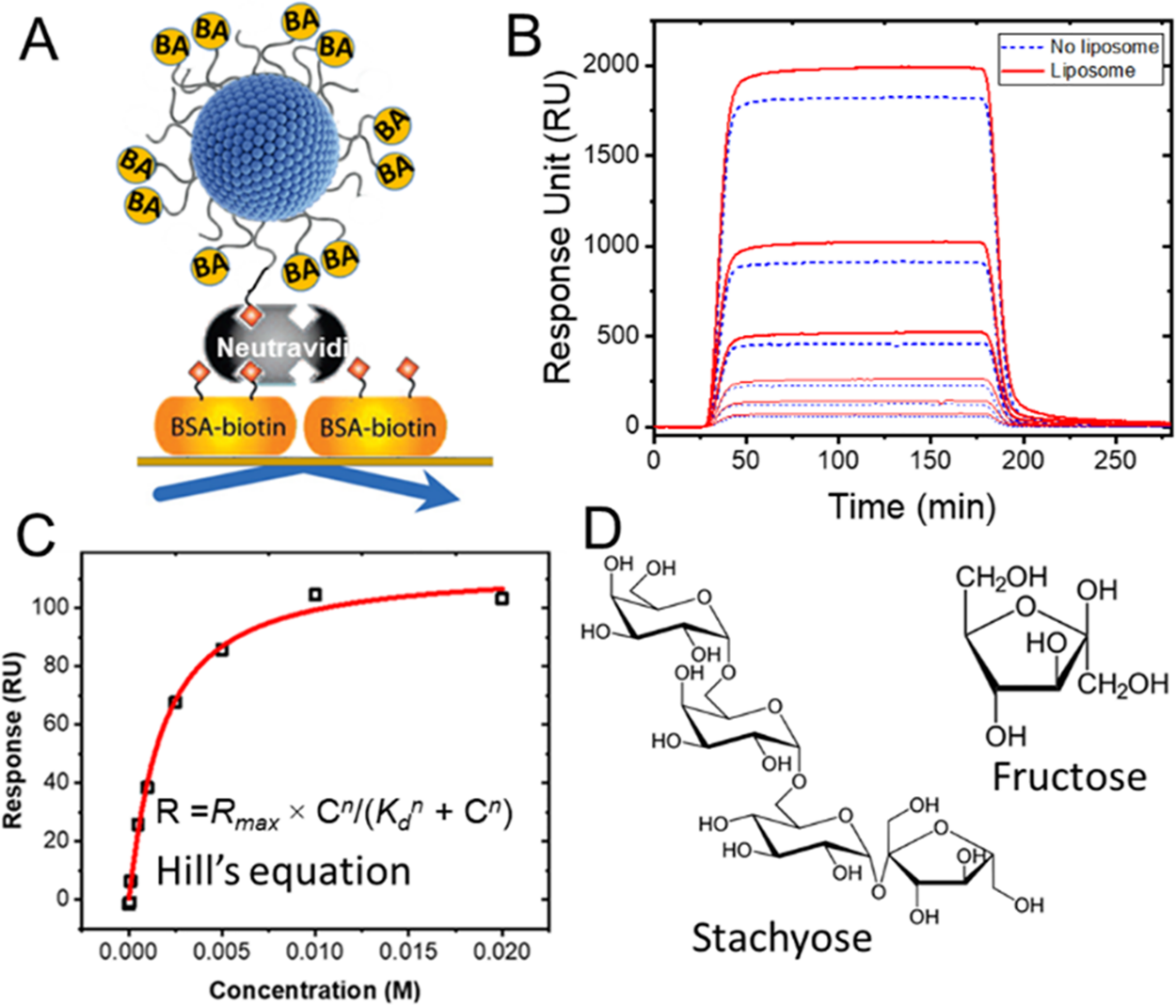
(A) Schematic representation of the immobilized liposomes on the SPR gold chip. Liposomes (100–200 nm) are functionalized with biotin, which is coupled to a biotinylated BSA through Neutravidin. (B) The SPR response upon injection of saccharides at different concentrations to the control (surface before liposome immobilization, dashed curves) and liposome channel (surface after liposome immobilization, solid curves). (C) The corrected SPR response corresponding to the interaction of a saccharide with BA-modified liposomes as a function of saccharide concentration. These values are the difference between the achieved constant level upon injection of saccharides at different concentrations and the control and liposome channels.

Surface plasmon resonance (SPR) spectroscopy was used to assess the binding affinity of saccharides to liposomes. We incorporated biotinylated lipids (DMPE-PEG-biotin) into the liposome formulations and immobilized them to a gold surface via a biotin–Neutravidin–biotin sandwich linkage (see Figure 2A). To assess saccharide binding, the respective SPR response of varying concentrations of saccharide over a surface containing no liposome was subtracted from that of a surface with liposomes (Figure 2B and C). A tetrasaccharide stachyose was employed as the imprinting template. The binding behavior of imprinted and nonimprinted liposomes was investigated using stachyose and the monosaccharide fructose as control (Figure 2D).

**Figure 3:**
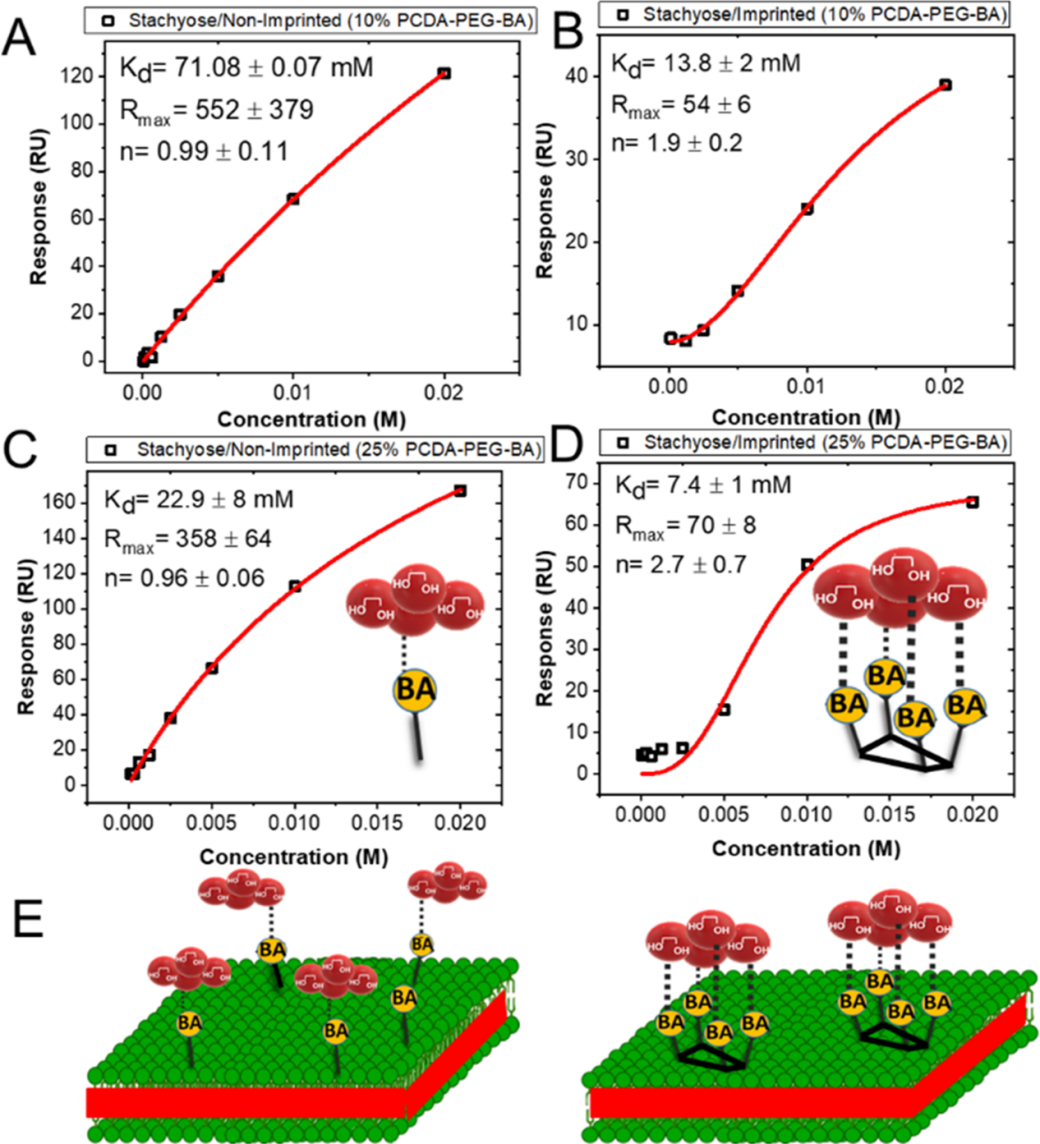
Binding curves corresponding to the interaction of tetrasaccharide stachyose and (A) nonimprinted and (B) imprinted liposomes composed of PCDA-PEG:PCDA-PEG-BA (10%). To generate imprinted liposomes, stachyose was used as the template. Binding curves of stachyose to (C) nonimprinted and (D) imprinted PCDA-PEG:PCDA-PEG-BA (25%) liposomes. (E) Schematic illustration of the effect of BA clustering on the binding behavior of the oligosaccharide. The formation of BA clusters reduces the number of binding sites accessible for binding but increases the binding strength due to multiple interactions.

The SPR responses to stachyose binding to the nonimprinted and imprinted liposomes composed of PCDA-PEG:PCDA-PEG-BA (10%) are shown in Figure 3A and B, respectively.

The SPR response (*R*) as a function of concentration (*C*) was fitted by the Hill equation,^39^*R* = *R*_max_ × C^*n*^/(*K*_d_^*n*^ + C^*n*^), where *R*_max_ is the SPR response unit (RU) at the saturation concentration, *K*_d_ is the RU value that gives 50% *R*_max_, which represents the apparent dissociation constant that reflects the affinity, and *n* is the Hill coefficient, which reflects the degree of cooperativity. This model is broadly used to quantify the affinity and cooperativity of multivalent interactions. The *K*_d_ of the stachyose, which was used as the template for imprinting, was more than 5-fold (13.8 ± 2 mM) lower than that of the nonimprinted liposomes (71.08 ± 0.07 mM) (Figure 3A and B), indicating that indeed the imprinting process resulted in a stronger affinity between BAs and the target saccharide. Notably, the Hill coefficient for the nonimprinted liposomes was close to 1 (*n* = 0.99 ± 0.11), indicating noncooperative binding, whereas the *n* value for the imprinted liposomes was greater than 1 (*n* = 1.9 ± 0.2), indicating positive cooperativity. Because the boronic acid concentration is the same in both formulations, the observed difference in *K*_d_ and *n* clearly indicates the formation of binding sites composed of several BA molecules and thus multivalent interaction.

The BA molecules in the binding cluster on the surface of the imprinted liposomes can be seen to interact sequentially with the four monosaccharide units that comprise stachyose. Thus, the observed positive cooperativity suggests that the interaction of individual monosaccharides facilitates the binding of the next unit. Cooperative binding of synthetic multivalent ligands at the lipid bilayer surfaces has previously been demonstrated.^15,40^ The BA molecules on the surface of nonimprinted liposomes are most likely distributed separately and homogeneously, allowing each tetrasaccharide to interact stably with only one BA molecule and thus no cooperativity.

To gain more insight about the organization of BAs within the clusters, we performed imprinting on liposomes containing a relatively high concentration of BAs (25%). The imprinting in this system also led to increased affinity (nearly 3-fold). Importantly, like the liposomes containing 10% BA, the Hill coefficient for imprinted liposomes became greater than 1 (*n* = 2.7 ± 0.7), while the *n* value for nonimprinted liposomes was close to 1 (*n* = 0.96 ± 0.06) as expected (Figure 3C and D). The results suggest that the local distribution of BA molecules in these liposomes was indeed impacted by imprinting. At this BA molar concentration, assuming that molecules are homogeneously dispersed prior to imprinting, in theory, each BA molecule is surrounded by three lipids that bear no BA. The fact that the imprinting process affected the BA distribution even at such low molecular spacing suggests that the BAs were patterned at the molecular scale, which further implies the formation of nanoclusters with nonrandom lateral organization of BAs.

While the apparent binding affinity of stachyose was higher for the imprinted liposomes, the *R*_max_ for the imprinted liposomes (70 ± 8 RU) was significantly lower than that of the nonimprinted liposomes (385 ± 65 RU). Because the concentration of BA in the two formulations is the same, this observation further points to a difference in the distribution of BA on the surface of liposomes. In contrast to the situation where BA molecules are not clustered, the formation of BA nanoclusters decreases the total number of individual binding sites that are available for binding (see Figure 3C). As a result, while the affinity for each BA cluster is increased due to the multivalent interaction, the total number of binding sites, and thus the *R*_max_, is expected to be reduced.

**Figure 4:**
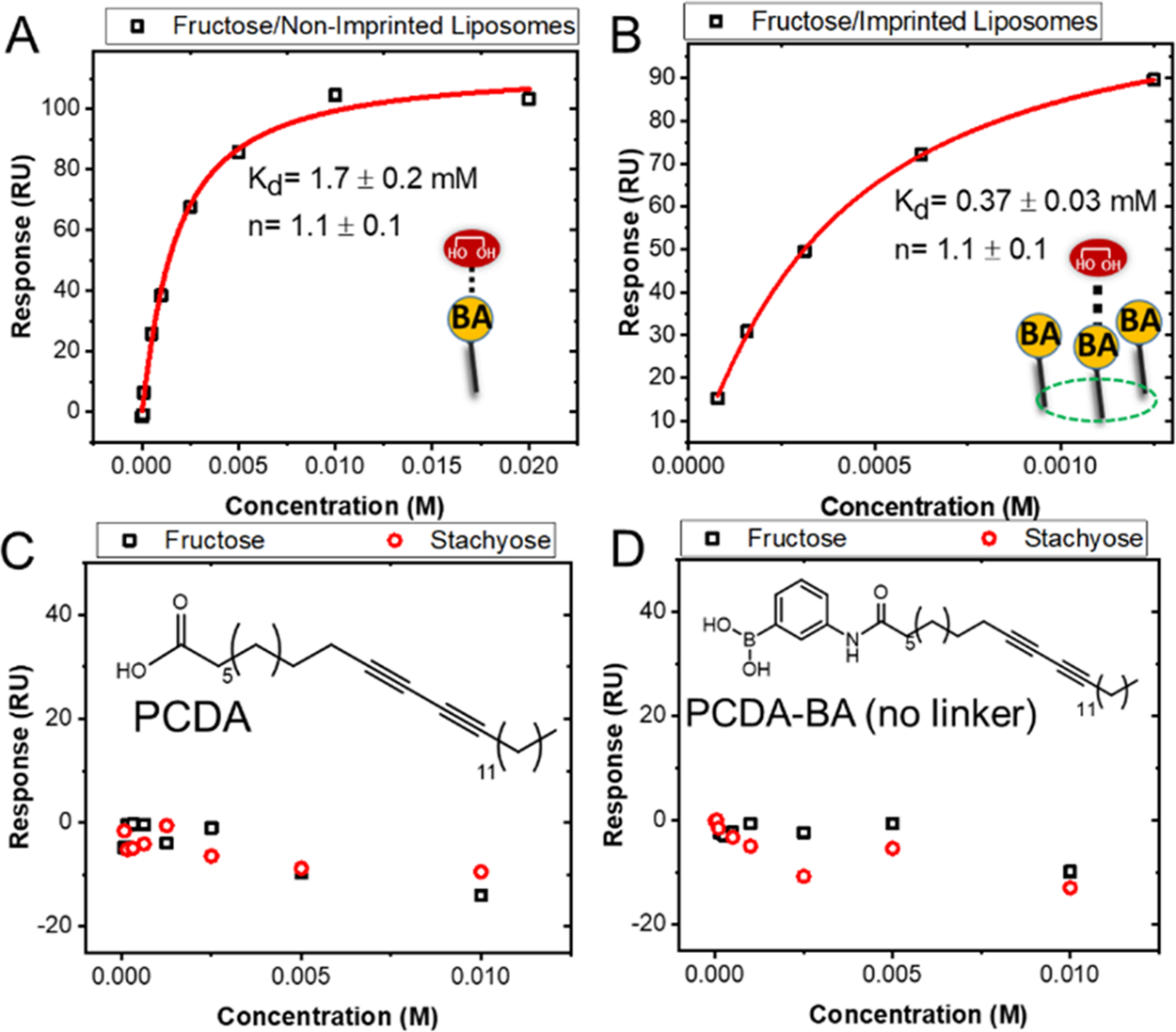
Binding curves corresponding to the interaction of monosaccharide fructose with (A) nonimprinted and (B) imprinted liposomes composed of PCDA-PEG:PCDA-PEG-BA (4:1). To generate imprinted liposomes, stachyose was used as the template. Binding curves corresponding to the interaction of stachyose and fructose with liposomes composed of (C) PCDA and (D) PCDA-PEG:PCDA-BA. No significant interaction was observed. The insets show the molecular structure of PCDA and PCDA-BA with no PEG linker.

The formation of BA nanoclusters on the surface of imprinted liposomes was then further investigated by measuring the interaction of a monosaccharide, fructose, with the imprinted and nonimprinted liposomes. Despite using stachyose as the template, fructose affinity for the imprinted liposomes was 6 times greater than that of the nonimprinted liposomes (Figure 4). The colocalization of BAs within each cluster raises their effective local concentration, which boosts the multivalency and hence affinity. However, in contrast to the stachyose binding, the Hill coefficient for the interaction of monosaccharide fructose with both imprinted and nonimprinted liposomes was close to 1, indicating a 1:1 binding stoichiometry with no cooperativity. Thus, even though the interaction of individual monosaccharide molecules with each BA cluster is stronger, they are not cooperative, as they interact independently.

To control the specificity of the interactions, we created liposomes made entirely of PCDA and devoid of BA. PCDA liposomes did not interact with stachyose or fructose, as expected (Figure 4C). We next prepared liposomes composed of PCDA-PEG:PCDA-BA, in which BA is connected to the PCDA directly without the use of a PEG linker. As shown in Figure 4D, no binding was observed in this formulation, indicating that the BAs are shielded by a PEG overbrush layer, which inhibits their exposure and reduces the accessibility to the saccharides. This further suggests that the binding observed for PCDA-PEG:PCDA-PEG-BA is specifically mediated by BA–saccharide interactions.

To study the possible effect of receptor nanoclustering on cell–cell interactions as well as nanoparticle drug delivery vehicles where both receptors and ligands are attached to the surface of interacting cells or nanoparticles, we investigated how BA nanoclustering affects the interaction of liposomes with surface-attached oligosaccharides rather than in-solution oligosaccharides. To this end, a biotinylated model trisaccharide (3′-sialyllactose-sp-biotin) was immobilized to a glass surface via a biotin-Neutravidin linker. The surface was coated with a passivating layer composed of PLL-*g*-PEG and PLL-*g*-PEG-biotin (10%).^41^ Such biotin concentration guarantees the high density of biotin-Neutravidin available for immobilization of densely packed 3′-sialyllactose.

**Figure 5:**
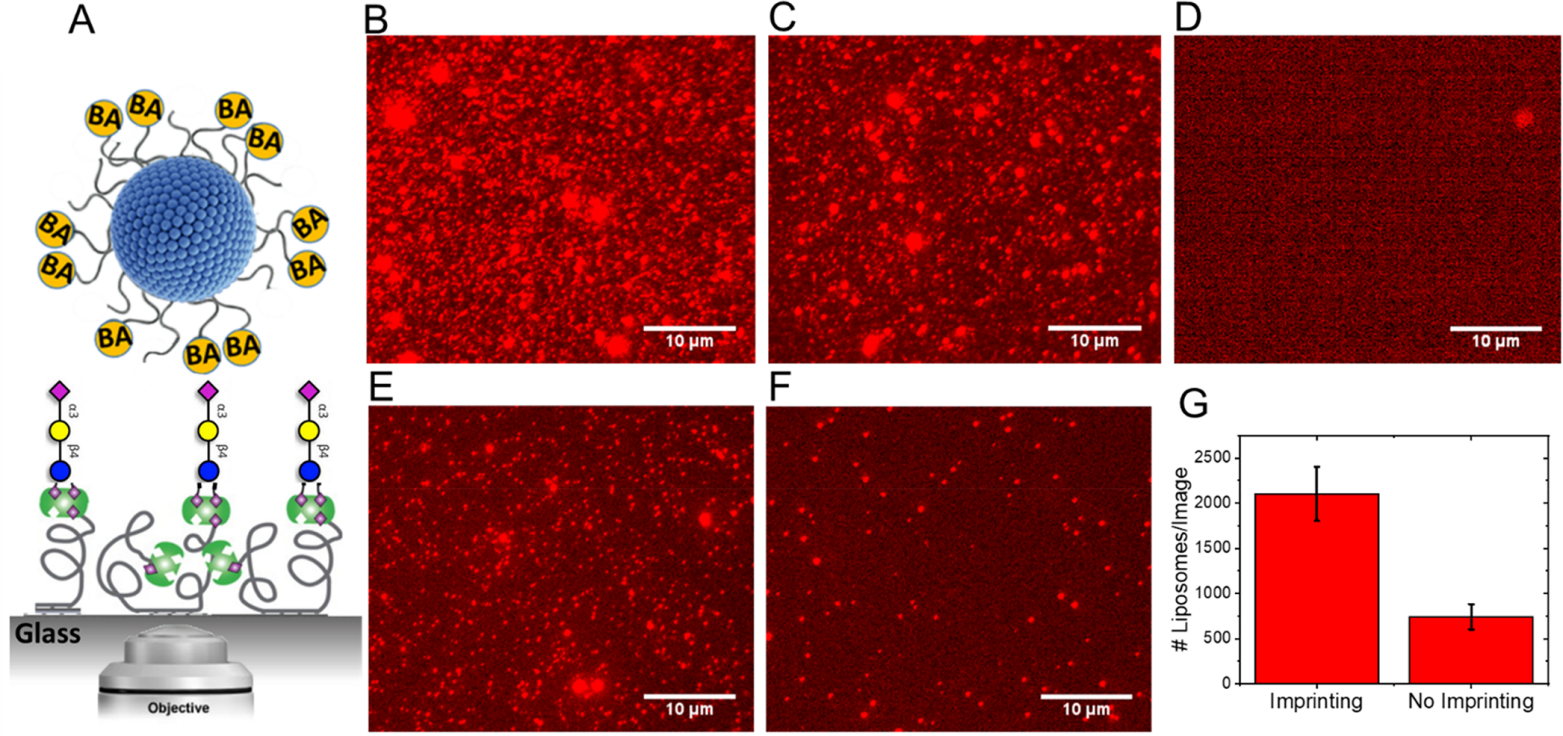
(A) Schematic depicting a fluorescence microscopy assay for detecting liposome–cell surface saccharide binding. Biotinylated 3′-sialyllactose molecules are attached to a PLL-*g*-PEG-biotin model surface through a biotin-Neutravidin linker. To assess the interaction of the liposomes with the surface-attached saccharides, imprinted and nonimprinted liposomes containing a trace amount of a fluorescently labeled lipid (Rhod-PE) were incubated with the surface in a microfluidic channel for a certain amount of time (10 min) and then washed to remove the unbounded liposomes. The bound liposomes were imaged by a fluorescence microscope. Representative fluorescence microscopy image of (B) imprinted and (C) nonimprinted liposomes composed of PCDA-PEG:PCDA-PEG-BA (25%) bound to the saccharide-decorated surface shown in (A). (D) No liposomes bound to the control surface with no 3′-sialyllactose. Fluorescence microscopy images of (E) imprinted and (F) nonimprinted PCDA-PEG:PCDA-PEG-BA (10%) liposomes. (G) Total number of bound liposomes per image for imprinted and nonimprinted liposomes. More representative images are presented in Supporting Figure S2.

Fluorescence microscopy was used to monitor the binding of the BA-functionalized liposomes to the surface (Figure 5A). To this end, a small amount of a fluorescently labeled lipid was added to the liposome formulation for imaging purposes. The imprinting was performed using 3′-sialyllactose as the template. Figure 5B and C show typical fluorescence images of surface-bound liposomes containing 25% BA for imprinted and nonimprinted liposomes, respectively. While the total number of imprinted liposomes bound to the surface was slightly higher than that of the nonimprinted liposomes, the difference was not statistically significant. To check if the binding was mediated by saccharides, the interaction between the liposomes and a surface with a similar coating but lacking 3′-sialyllactose was evaluated. As expected, no binding was detected (Figure 5D).

The observation that imprinting did not improve the affinity toward densely packed surface-attached oligosaccharides indicates that the BA organization at the liposomes’ surface had no impact on the interaction, at least at this BA density (25%). This suggests that even if the BAs are randomly distributed, liposomes can interact with multiple units of the densely packed surface-attached saccharides, provided that the BA surface density is high enough. To confirm this, we next tested the binding interaction of imprinted and nonimprinted liposomes containing a lower concentration of BA (10%) (Figure 5E and F). The number of bound imprinted liposomes in a typical image was significantly higher than that of nonimprinted liposomes at this BA concentration (Figure 5G). Generally, multivalent binders have a high probability of rebinding with either the same site or another one in close proximity, leading to extremely long residence times and potentially irreversible interactions.^42^

Taken together, contrary to the behavior of individual in-solution saccharides, the interaction between surface-attached saccharides and the liposomes is dependent on the BA density. Since the surface is fully covered by the oligosaccharides, the chance of multiple engagement between the liposomes with high surface densities of BA and the surface-attached saccharides is high; therefore, the local organization of BAs is less important. However, at lower BA surface densities, the likelihood of multivalent interactions between liposomes and surface-attached saccharides is highly dependent on BA organization.

Next, we studied the effect of receptor crowding. To create a model that demonstrates receptor crowding, domains of densely packed BA amphiphiles must be generated. To accomplish this, we utilized membrane phase separation, the process by which lipid membranes containing multiple types of lipids separate into domains with different lipid compositions based on the physical and chemical characteristics of their head groups and hydrocarbon tail groups under specific conditions.^43^

**Figure 6:**
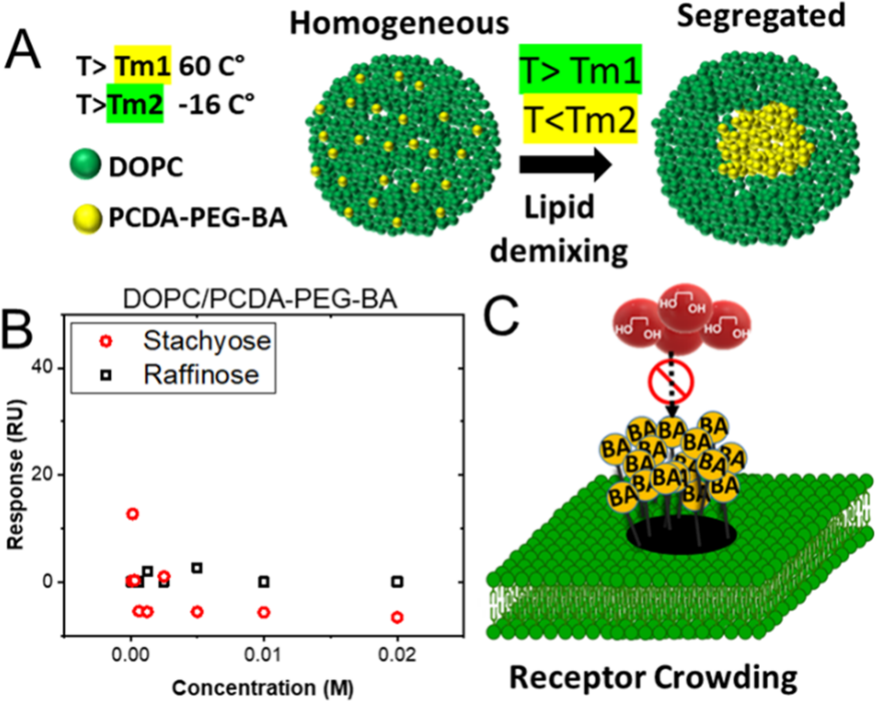
(A) Schematic representation of the phase separation process in the liposomal membrane of a binary lipid mixture with different *T*_m_. Lipids are homogeneously mixed at a temperature above the *T*_m_ of both lipids. Lipids undergo demixing upon cooling to ambient temperature, resulting in the formation of domains of distinct lipids. (B) SPR data corresponding to the interaction of tetrasaccharide stachyose and trisaccharide raffinose with liposomes composed of DOPC/PCDA-PEG-BA (25%). No significant interaction was observed. (C) Schematic illustration of receptor crowding and nanoclustering. When BA molecules are densely and randomly packed, they obstruct ligand binding, presumably due to the steric effect.

In general, binary lipid systems containing a lipid with a gel to liquid transition temperature well below room temperature and a lipid with a transition temperature well above room temperature tend to phase separate into two domains at room temperature (Figure 6A):^44^ one domain with a solid ordered phase primarily containing lipids with high *T*_m_ and one domain with a liquid disordered phase primarily containing lipids with low *T*_m_. It has been shown that when photopolymerizable diacetylene lipids (*T*_m_ > 60 °C) are mixed with unsaturated phospholipids such as DOPC (*T*_m_ = −16 C), they phase separate when the system is cooled from a temperature higher than the diacetylene lipid’s *T*_m_ to room temperature.^45^ Accordingly, we prepared liposomes composed of DOPC and PCDA-PEG-BA (25%) at temperatures above the PCDA transition temperature (70 °C) and then gradually cooled the solution to room temperature (22 °C).

No binding was observed when the interaction of stachyose with these liposomes was monitored using SPR (Figure 6B). We also tested the interaction of another oligosaccharide, raffinose, and found no binding. The fact that even in the presence of 25% BA, no binding was observed indicates that indeed the packing of BAs can affect their presentation and may make the receptors inaccessible within the crowded domains.^46,47^ To verify that the receptor crowding is a result of phase separation due to the immiscibility of DOPC and PCDA-PEG-BA, we performed a FRET experiment, which is commonly used to examine lipid phase separation and nanodomain formation.^48,49^ A FRET pair, CF-PE as donor and Rh-PE as acceptor dye, with the Förster distance of ∼6.0–6.9 nm^50^ was included in the liposome formulation. FRET was measured at temperatures above and below the phase transition temperature of PCDA (∼60 °C). At room temperature, the FRET efficiency (*I*_Rh_/*I*_CF_ = 2.6) was significantly higher than that measured at a temperature greater than 60 °C (*I*_Rh_/*I*_CF_ = 1.4) (see Supporting Figure S3). Due to the alkyl chain structure of both FRET pairs, they are preferentially distributed in the DOPC-rich phase of lipid bilayers.^51^ The observed increase in FRET at room temperature consequently reveals that CF-PE and Rh-PE are concentrated in the DOPC-rich phase, as a result of phase separation between PCDA-PEG-BA and DOPC. To confirm the presence of boronic acids at the outer leaflet of the liposomes after incubation at a high temperature, we conducted an Alizarin Red S (ARS) assay.^52^ ARS itself is not a fluorescence-active compound, but upon ester formation with the boronic acid, the ARS adduct becomes fluorescent.^53^ Upon incubation of liposomes with ARS, there was a notable increase in the fluorescence intensity of ARS that was proportional to the liposome concentration (see Supporting Figure S4). The results indicate that BA is present in the other leaflet of the liposomes, since ARS, being a water-soluble agent, is unable to traverse the liposomal bilayer. Taken together, while the template-guided imprinting resulted in the formation of an ordered organization of BAs resembling cell surface nanoclusters, phase separation caused the BAs to cluster randomly, reminiscent of receptor overexpression and crowding (Figure 6C).

## Conclusion

In summary, in this work we have demonstrated that not only the surface density but the arrangement of the receptors at the nanoscale have a great impact on the ligand binding at the membrane interface. To this end, we explored the interactions between multivalent ligands and prearranged nanoclusters as well as the crowded domains of a model membrane-embedded receptor. Using photopolymerizable lipids to form the liposomes, it was possible to “fix” lipids in the liposome membrane by UV irradiation and, in so doing, capture the spatial organization of the BA lipids that have been directed by the binding of a template saccharide. Even weak interaction between an oligosaccharide and membrane-embedded BAs can organize them into nanoclusters. The implication is that for membrane-embedded receptors even low-affinity multivalent interaction can lead to the organization of membrane receptors as seen in numerous examples in biology such as the activation of adaptive immune cells,^54,55^ the formation of stable focal adhesions,^56,57^ and pathogen attack.^58^

When compared to randomly distributed BAs, controlled nanoclustering of BAs on the lipid membrane surface increased the binding affinity by a factor of 5. The finding that the cooperativity of the interaction is very sensitive to the nanoscale arrangement of membrane receptors is consistent with the attribution of highly sensitive biological response to the presence of ordered receptor nanoclusters. For instance, it has been demonstrated that TCRs are frequently present in the form of linear nanoclusters with a diameter of approximately 10 nm in non-raft membranes that exist independently and before antigen binding.

The binding of oligosaccharide to domains of densely packed BAs was significantly hindered, presumably due to steric hindrance. The findings suggest that nanoclusters have a high degree of lateral organization, distinguishing them from densely packed, crowded clusters. Receptor crowding hinders the accessibility of the receptors, posing a potential challenge for the targeted delivery of therapeutic agents to overexpressed cell-surface molecular markers. Such a mechanism has been proposed for the resistance to antibody therapy in tumors with very high levels of the target membrane receptor.^59^

The surface imprinting approach allows for a finely controlled arrangement of BA units, which provides a path for the selective and sensitive targeting of glycans. Given that only one type of recognition moiety (i.e., BA) was used in this work, the results suggest that the performance of imprinted liposomes can be improved, and the extension of the liposome surface imprinting process to more complex systems can be readily envisaged. Taken together, our results imply that the nano-organization and expression density of cell membrane receptors can finely tune the dynamics of biological ligand–receptor interactions as well as receptor accessibility, posing a potential challenge for the targeted delivery of therapeutic agents to overexpressed cell-surface molecular markers. Finally, the strategies developed in this work can be applied to form and study other customized nanoclusters composed of amphiphilic peptides, glycans, or any bioactive epitopes, in which lateral organization determines function.

## Experimental Section

### Liposome Preparation and Imprinting

A general procedure for the preparation of PDA liposomes involved first dissolving the PDA monomers, along with any other lipid constituents desired, in chloroform to generate a homogeneous distribution of monomers. Chloroform was then evaporated, by a N_2_ stream. The dried lipid film was kept under vacuum for at least 3 h, after which it was resuspended in deionized water or aqueous buffer (0.1 M ammonium acetate at pH 10) to achieve a total lipid concentration of 1 mM. After vortex mixing, the resulting suspension was dispersed by sonication at 80 °C for 30 min. After the sonication process, the liposome solution was immediately filtered using a disposable 0.45 μm syringe filter to remove aggregated material or large particles. For molecular imprinting, stachyose or 3′-sialyllactose solution was added to the liposome suspension to achieve a final saccharide concentration of 20 M and further incubated at 80 °C for 1 h. After this, the solution was slowly cooled and stored at 4 °C for 12 h. Polymerization of the liposome solution was carried out by irradiating the solution with a UV lamp (254 nm) for 10 min. The same procedure was used for preparation of nonimprinted liposomes except no saccharide was added to the liposome suspension before the cooling and polymerization steps.

### Surface Plasmon Resonance

SPR experiments were performed with a Reichert SR7000DC dual channel spectrometer at 25 °C. Flow rate for all experiments was 25 μL/min. The gold substrates were cleaned by immersion in piranha solution (70% H_2_SO_4_, 30% H_2_O_2_) for 10 min at room temperature and rinsed with Milli-Q water and HPLC grade ethanol. (**Caution**: *Piranha solution reacts violently with all organic compounds and should be handled with care*.) Clean substrates were installed inside the SPR spectrometer, and a degassed 1× PBS pH 7.4 solution was run over it until a stable baseline was achieved. A PBS solution of BSA–biotin (0.25 mg/mL, 10 min) was injected next, reaching a response ranging from 300 to 500 RU, followed by 10 min of dissociation in the running buffer. Just after, Neutravidin (0.1 mg/mL, 10 min, in PBS pH 7.4) was injected, reaching a response in the range 2000–2500 RU, followed by 10 min of dissociation in the running buffer. At this point, the running buffer was changed to the one liposomes were made on (ammonium acetate pH 10, 0.1M). Once the baseline is stable, solutions (0.01–20 mM) of saccharides were injected during 2.5 min each, followed by 3 min of dissociation in the running buffer. After 15 min, the liposome solution (as prepared) was injected for 10 min, followed by 1 h of dissociation in the running buffer. At this point, all previous saccharide injections were repeated exactly. SPR sensograms were processed using Scrubber 2 software. Definitive SPR data were obtained after subtracting the bulk refractive index steady-state response of saccharides from the corresponding steady-state response of the surface containing the saccharide-binding liposomes.

### Fluorescence Microscopy

Glass microscope coverslips were first cleaned by SDS (1%), treated by UV ozone for 20 min, and then assembled into a commercially available six-channel flow cell (μ-Slide VI 0.4, Martinsreid, Germany) of rectangular geometry with a height of 0.4 mm and a total volume of ∼60 μL. The coverslips were coated by a self-assembled monolayer of a 10:1 mixture of poly(l-lysine)-grafted poly(ethylene glycol) (PLL-*g*-PEG) and PLL-*g*-PEG(-biotin) (SuSoS AG). The PEGylated surface was further incubated with Neutravidin (10 μg/mL) for 20 min and subsequently washed carefully. Biotinylated oligosaccharide (3′-sialyllactose-sp-biotin, CAS: 1384441-58-0) was immobilized at a concentration of 0.1 μg/mL for 30 min. The liposome membrane contained 0.5 wt % rhodamine-PE. A liposomes solution of 0.1 mg/mL was injected into the channel and incubated for 10 min before washing thoroughly with PBS buffer to remove unbonded liposomes. Liposomes were subsequently imaged using an inverted Eclipse TE 2000 microscope (Nikon) equipped with a high-pressure mercury lamp, a 60× oil TIRF objective (NA 1.49), and an Andor iXon+ EMCCD camera (Andor Technology, Belfast, Northern Ireland). Images were taken from different parts from three channels for each measurement using the Nikon NIS-Elements platform with the following acquisition parameters: binning: 2 × 2, exposure: 200 ms, readout mode: rolling shutter at 16-bit, readout rate: 560 MHz. Images were analyzed by ImageJ. Briefly, images with .nd2 format were converted to TIF files using ImageJ ND2 Reader plugin. The Analyze Particles function in ImageJ was used to count fluorescent particles (liposomes) in each image; the image threshold was set to isolate the fluorescent particles (Image > Adjust > Threshold) and then the number of particles was measured using Analyze Particles (Analyze > Analyze Particles). In the Analyze Particles dialogue box the following settings were used: size: 0–infinity, circularity: 0–1. All measurements were performed at room temperature (*T* = 22 °C). The average size of liposomes was ∼200 ± 59.9 nm, which was measured by dynamic light scattering (DLS, Figure S1).

### FRET

The phase separation of PCDA-PEG-BA and DOPC was examined by the FRET assay; 18:1 PE-carboxy fluorescein (CF-PE) and 18:1 PE-rhodamine (Rhod-PE) were included in the liposome formulation at 0.25 mol %. The fluorescence intensity was monitored with a fluorescence spectrometer (spectrofluorometer; Edinburgh Instruments FS5). The emission spectra (500 to 700 nm) were collected upon excitation at 490 nm (excitation wavelength of CF-PE). As an index of the FRET efficiency, the intensity ratio (Ir) was defined as Ir = *I*_Rh_/*I*_CF_. When the phase separation occurs, CF-PE and Rh-PE are both partitioned into DOPC domains and Ir increases (Figure S3).

### Alizarin Red S Assay

The fluorescence spectra of solutions containing a constant amount of ARS (9 μM) but varying concentrations of liposomes composed of DOPC/PCDA-PEG-BA (25%) were measured using a fluorescence spectrometer (spectrofluorometer; Edinburgh Instruments FS5), with the samples being excited at 466 nm.

## Supporting Information Available

Supplementary figures and characterization data; additional details about the synthesis procedures (PDF)
