# Supplementary material for: Recapitulating the Lateral Organization of Membrane Receptors at the Nanoscale
Source: ACS Nano. 2023 May 18;17(11):10327–36. doi: 10.1021/acsnano.3c00683 (PMC10278181; doi:10.1021/acsnano.3c00683)
Supplement: Supplementary file 1 — nn3c00683_si_001.pdf [file nn3c00683_si_001.pdf]

Recapitulating the Lateral Organization of Membrane Receptors at the Nanoscale

Seyed R. Tabaei[*†], Marcos Fernandez-Villamarin[§], Setareh Vafaei[§], Lorcan Rooney[†] Paula M. Mendes[§]

†School of Chemistry and Chemical Engineering, Queen's University Belfast, Stranmillis Rd, Belfast, BT9 5AG, UK

§School of Chemical Engineering, University of Birmingham, Edgbaston, Birmingham, B15 2TT, UK

General Materials and Methods

10,12-pentacosadiynoic acid and 3-aminophenylboronic acid were purchased from Alfa Aesar. 1,11-amino-3,6,9-trioxyundecane was purchased from Tokyo Chemical Industry. 3-carboxyphenylboronic acid was purchased from Acros organics. Poly(l-lysine)-grafted poly(ethylene glycol) (PLL-g-PEG) and PLL-g-PEG(-biotin) (PLL-g-PEG and PLL-g-PEG-biotin were purchased from SuSoS AG. 1,2-dioleoyl-sn-glycero-3-phosphoethanolamine-N-(lissamine rhodamine B sulfonyl) (ammonium salt) and 1,2-dioleoyl-sn-glycero-3-phosphoethanolamine-N-(carboxyfluorescein) (ammonium salt) were purchased from Avanti Polar Lipids. NeutrAvidin and biotinylated bovine serum albumin (B-BSA) were obtained from ThermoFisher Scientific. All other chemicals including Alizarin Red S were purchased from Sigma-Aldrich. Gold substrates employed in SPR experiments were purchased from Reichert Technologies/Ametek Inc. Ultrapurified water in all experiments was supplied by a Milli-Q water system (Millipore, Billerica, MA). Thin layer chromatography was carried out on Merck-Millipore Silica gel 60 F_{254} aluminium sheets and visualised using 254 nm UV light or curcumin stain solution. Silica gel chromatography was performed using Sigma-Aldrich (pore size 60 Å, 40-63 µm particle size) silica gel. ^1H NMR spectra were recorded on a 400 MHz Bruker AVANCE NEO 400 NMR spectrometer at room temperature. Mass spectra were recorded with Waters Xevo G2-XS using electrospray ionization mode.

Synthesis

Synthesis of PCDA-PEG-NH_2

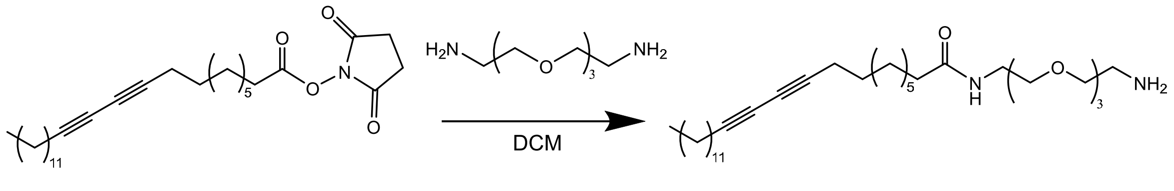

1.01g (2.15 mmol) of N-succinimidyl-10,12-pentacosadiynoate, prepared as described in reported procedure,[1] were dissolved in CH_2Cl_2 (20 ml). Previous solution was slowly added over a solution of 1,11-amino-3,6,9-trioxyundecane (2.06 g, 10.74 mmol) in CH_2Cl_2 (52 ml). After being stirred for 1h at room temperature, reaction mixture was evaporated, redissolved in EtOAc and washed with water twice, dried with $MgSO_4$, and evaporated. After being purified by silica gel chromatography ($CHCl_3$ to $CHCl_3$/MeOH 10%), final product (1.00g, 85%) was obtained as a white solid.

[1] J. Am. Chem. Soc., 1993, 115(3), 1146-1147, DOI: 10.1021/ja00056a047

^{1}H NMR (400 MHz, $CDCl_3$) δ 3.68 - 3.59 (m, 8H), 3.59 - 3.52 (m, 4H), 3.45 (q, *J* = 5.3 Hz, 2H), 2.90 (t, *J* = 5.1 Hz, 2H), 2.24 (t, *J* = 7.0 Hz, 4H), 2.18 (t, *J* = 7.5 Hz, 2H), 1.62 (p, *J* = 7.5 Hz, 2H), 1.56 - 1.45 (m, 4H), 1.44 - 1.18 (m, 27H), 0.87 (t, *J* = 6.9 Hz, 3H). ESI MS m/z: 594.46 Calcd. for $C_{33}H_{61}N_2O_4$; 549.46 Found $[M+H]^+$.

Synthesis of PCDA-PEG-BA

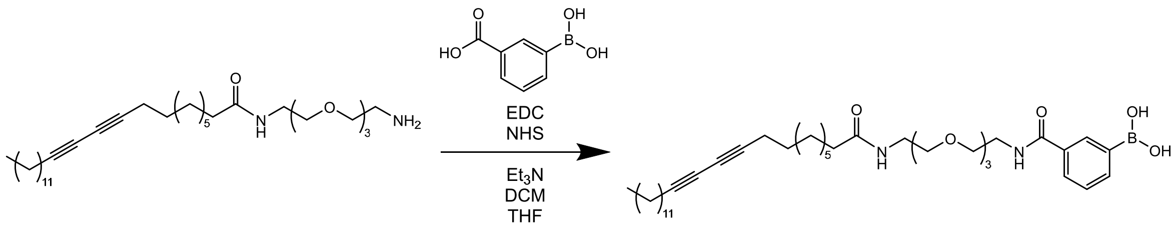

0.97g (1.77 mmol) of PCDA-PEG-NH_2 were dissolved in CH_2Cl_2 (12.5 ml) and THF (12.5 ml). Then, 3-carboxyphenylboronic acid (0.44g, 2.65 mmol), Et_3N (0.49 ml, 3.53 mmol), NHS (0.37 g, 3.18 mmol) and EDC (0.61 g, 3.18 mmol) were added sequentially. After being stirred overnight at room temperature, reaction mixture was evaporated, dissolved in $CHCl_3$ and washed with water and brine, dried with $MgSO_4$ and evaporated. After being purified by silica gel chromatography (EtOAc/MeOH 2% to EtOAc/MeOH 10%, then $CHCl_3$/MeOH 10%), final product (0.72g, 59%) was obtained as a clear oil after evaporation.

^{1}H NMR (400 MHz, $CDCl_3$) δ 8.19 (s, 1H), 8.03 (d, *J* = 7.9 Hz, 1H), 7.97 (d, *J* = 7.4 Hz, 1H), 7.48 (t, *J* = 7.6 Hz, 1H), 3.73 - 3.58 (m, 8H), 3.53 (t, *J* = 5.5 Hz, 2H), 3.38 (q, *J* = 5.5 Hz, 2H), 2.23 (t, *J* = 6.9 Hz, 4H), 2.18 2.14 (m, *J* = 7.6 Hz, 2H), 1.50 (q, *J* = 7.2 Hz, 4H), 1.43 - 1.18 (m, 26H), 0.87 (t, *J* = 6.8 Hz, 3H). ESI MS m/z: 697.50 Calcd. for $C_{40}H_{66}BN_2O_7$; 697.50 Found $[M+H]^+$.

Synthesis of PCDA-BA

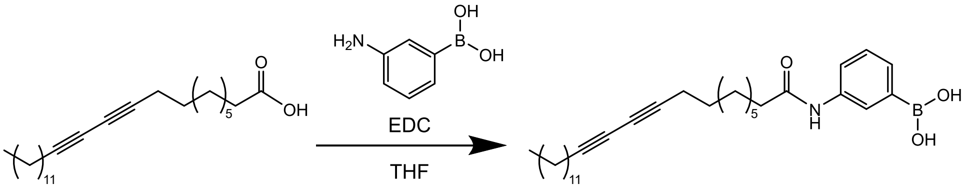

0.50 g (1.33 mmol) of 10,12-pentacosadiynoic acid were dissolved in THF (18 ml). Then 3-aminophenylboronic acid (0.27g, 2.00 mmol), and EDC (0.46 g, 2.40 mmol) were added sequentially. After being stirred overnight at room temperature, reaction mixture was evaporated under reduced pressure and purified by chromatography silica gel (EtOAc/MeOH 2%). Final product (0.16g, 24%) was obtained as a clear oil after evaporation.

^{1}H NMR (400 MHz, DMSO-d_6) δ 7.98 (s, 2H), 7.82 (s, 1H), 7.71 (d, *J* = 8.0 Hz, 1H), 7.45 (d, *J* = 7.1 Hz, 1H), 7.24 (t, *J* = 7.7 Hz, 1H), 2.30 - 2.24 (m, 6H), 1.72 - 1.63 (m, 2H), 1.62 - 1.53 (m, 4H), 1.49 - 1.40 (m, 26H), 0.86 (t, *J* = 6.7 Hz, 3H). ESI MS m/z: 494.38 Calcd. for $C_{31}H_{49}BNO_3$; 494.38 Found $[M+H]^+$.

Synthesis of PCDA-PEG

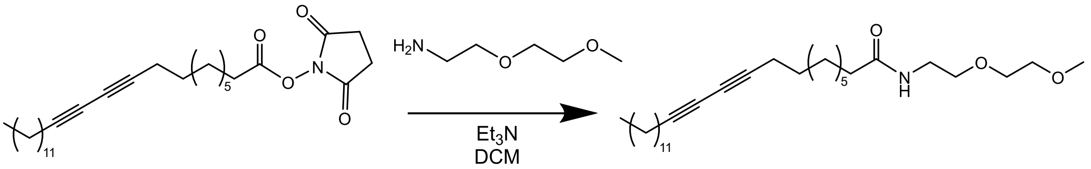

0.13g (0.3 mmol) of N-succinimidyl-10,12-pentacosadiynoate[1] were dissolved in CH_2Cl_2 (5.4 ml). Then (2-(2-methoxyethoxy)ethyl)amine (55.0 mg, 0.46 mmol) and Et_3N (0.1 mL, 0.69 mmol) were added sequentially. After being stirred for 3h at room temperature, reaction mixture was evaporated under reduced pressure and purified by silica gel chromatography in (CH_2Cl_2/EtOAc 50%). Final product (106 mg, 82%) was obtained as a white solid after evaporation.

^{1}H NMR (400 MHz, $CDCl_3$) δ 3.63 - 3.57 (m, 2H), 3.57 - 3.50 (m, 4H), 3.48 - 3.40 (m, 2H), 3.38 (s, 3H), 2.21 (t, *J* = 7.0 Hz, 4H), 2.15 (t, *J* = 7.2 Hz, 2H), 1.60 (p, *J* = 7.2 Hz, 2H), 1.49 (p, *J* = 7.9, 7.0 Hz, 4H), 1.43 - 1.19 (m, 26H), 0.86 (t, *J* = 6.7 Hz, 3H). ESI MS m/z: 476.41 Calcd. for $C_{30}H_{54}NO_3$; 476.41 Found $[M+H]^+$.

Supporting Figures

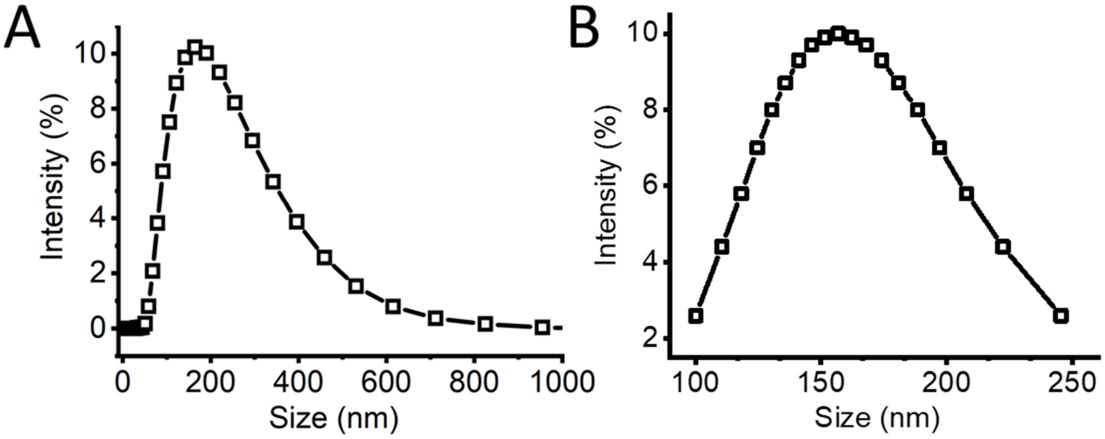

Figure S1. (A) DLS data indicating the average size distribution of imprinted liposomes after preparation. (B) DLS data of imprinted liposomes used for microscopy. Before microscopy, the liposome solution was filtered using a disposable 0.2 µm syringe filter to remove aggregated material or large particles.

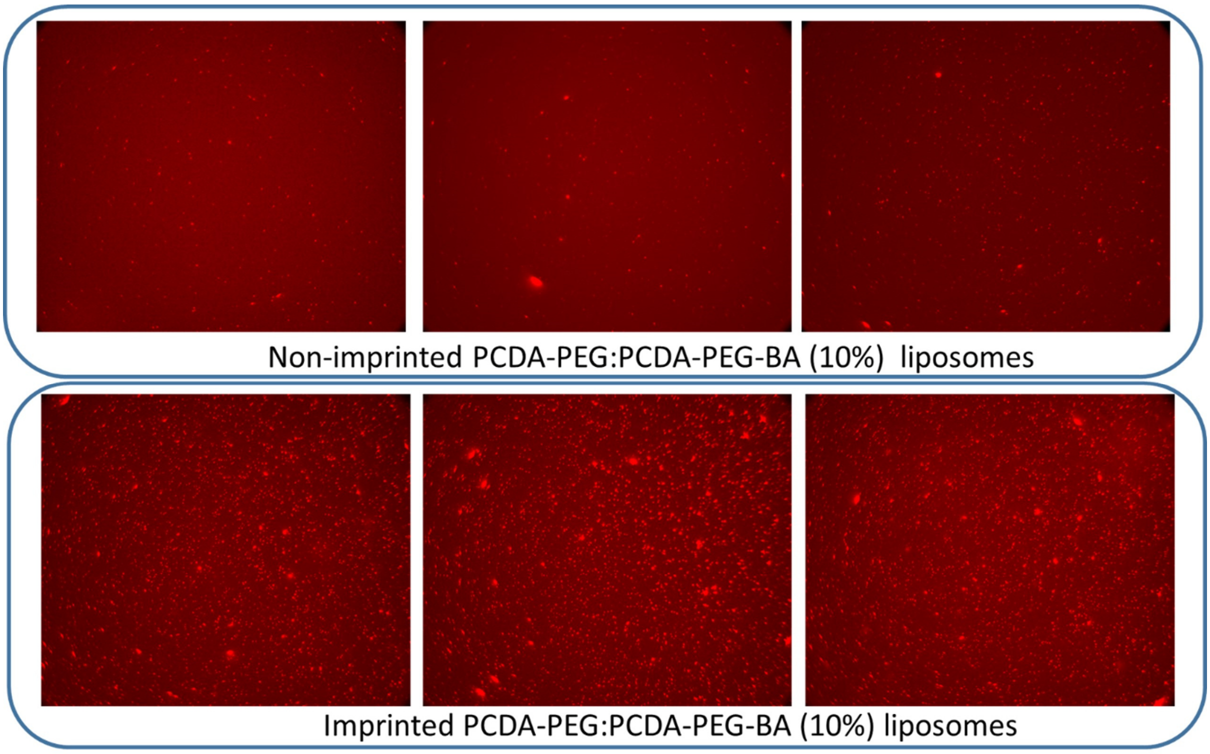

Figure S2. Representative fluorescence microscopy images show a comparison between non-imprinted (top row) and imprinted liposomes (bottom row) composed of PCDA-PEG:PCDA-PEG-BA (10%). The number of imprinted liposomes bound to the saccharide-decorated surface is significantly higher than that of the non-imprinted liposomes.

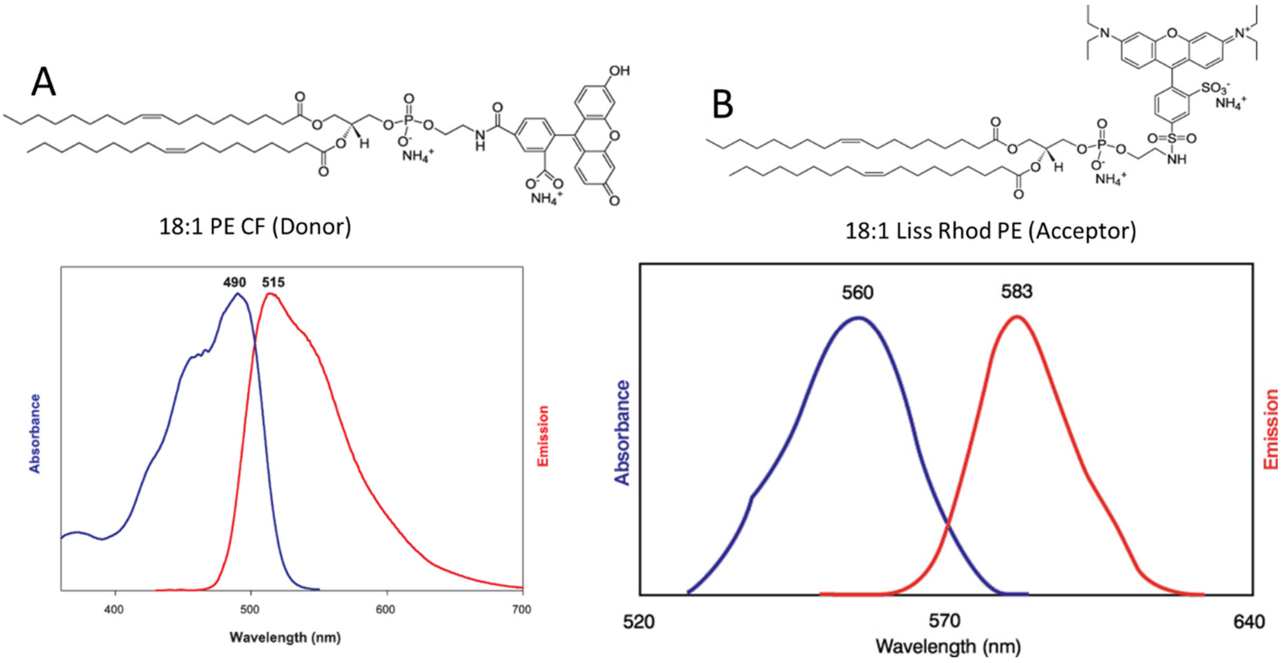

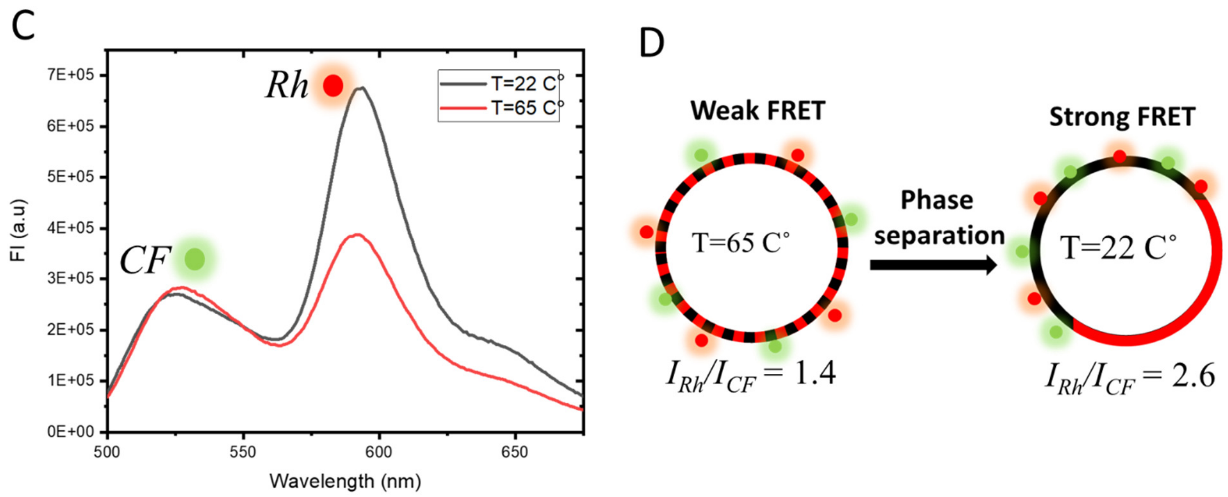

Figure S3. Molecular structure and absorption (blue line) and emission spectra (red line) A) CF-PE and , B) Rh-PE which were used as FRET donor and acceptor respectively, C) FRET results using Rh-PE as acceptor upon excitation at 490 nm at T= 65 C° (red line) and T= 22 C° (black line), D) Schematics of the FRET interaction between a CF-PE and Rh-PE upon lipid phase separation.

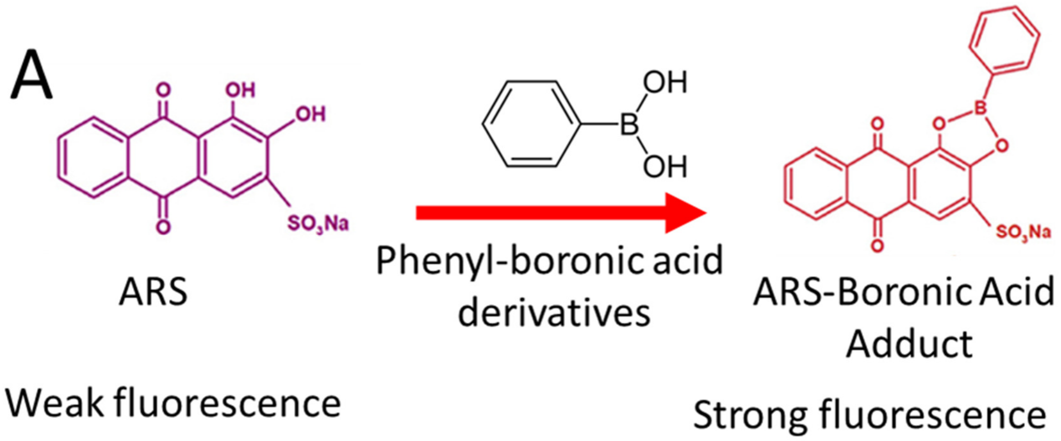

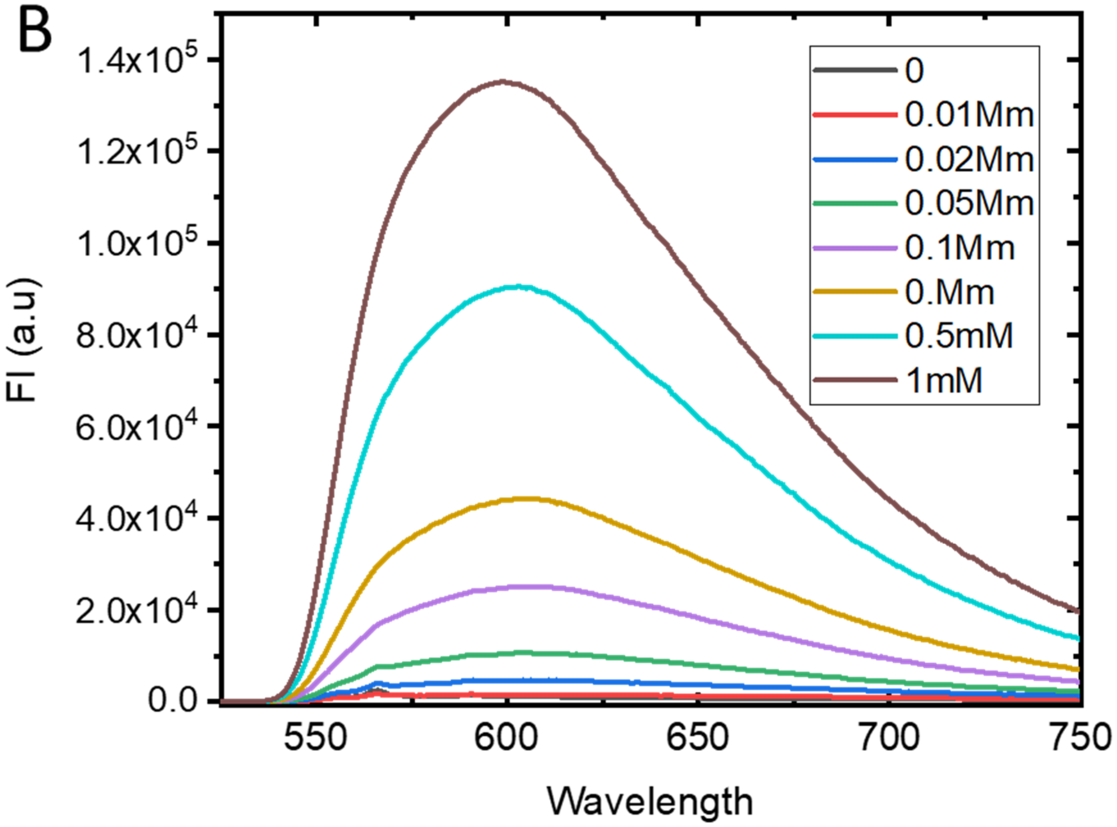

Figure S4. A) Representation of boronic acid-ARS reaction. ARS itself is not a fluorescence active compound but upon ester formation with the boronic acid, the ARS-adduct becomes fluorescent. B) Titration of DOPC/PCDA-PEG-BA (25%) liposome into a solution of ARS (10^{-6} M). Fluorescence increases with added liposomes (0 through 1 mg/ml), Exc. λ = 466 nm, Em. λ = 601 nm.
